# Contraction of the Uterine Orifices in Relation to Dysmenorrhea and Sterility

**Published:** 1881-02

**Authors:** 


Contraction of the Uterine Orifices in Relation to Dysmenorrhea and Sterility.—Pajot contributes to the December number of the *Annales de Gynecologie* a paper with the above title. His conclusions are the following: As all obstacles to the ready flow of the menstrual blood, contraction of the canal may be one of the causes of dysmenorrhea, but not an inevitable one.

When the physician believes this to be the cause, it is necessary to enlarge the two orifices.

Such enlargement by incisions exposes the patient to grave consequences and even to death.

Plugs, tents, dilating bodies to be retained, sometimes also cause very serious accidents.

The dilatation by graduated dilators, managed prudently and only used for a few minutes at each *seance*, have, on the contrary, so far proved absolutely inoffensive.

The external orifice only need be dilated for sterility consequent upon contraction.

In this last case the dilatory action exercised transversely appears to produce the condition more favorable to fecundation than circular dilatation.—*Amer. Practitioner.*

